# Clinical Efficacy of Tonic Traditional Chinese Medicine Injection on Acute Cerebral Infarction: A Bayesian Network Meta-Analysis

**DOI:** 10.1155/2020/8318792

**Published:** 2020-11-23

**Authors:** Dongrui Zhou, Liandi Xie, Yao Wang, Shuang Wu, Fengzhi Liu, Shuangshuang Zhang, Ruijia Liu, Lingqun Zhu

**Affiliations:** ^1^Key Laboratory of Chinese Internal Medicine of Educational Ministry and Beijing, Dongzhimen Hospital, Beijing University of Chinese Medicine, Beijing, China; ^2^Department of Cardiology, Dongfang Hospital, Beijing University of Chinese Medicine, Beijing, China; ^3^Department of Cardiology, Zhuji Hospital of Traditional Chinese Medicine, Shaoxing, Zhejiang, China; ^4^Department of Oncology, Beijing Daxing District Hospital of Integrated Chinese and Western Medicine, Beijing, China

## Abstract

Western medicine (WM) has certain limitations in terms of treating acute cerebral infarction (ACI), while tonic traditional Chinese medicine injections (TCMIs) have been shown to have obvious clinical effects as an adjunct to WM for ACI. However, most randomized controlled trials (RCTs) to date have not performed direct comparisons of efficacy among tonic TCMIs. This study designed a Bayesian network meta-analysis (NMA) to explore the therapeutic effect of tonic TCMIs on ACI. A comprehensive search of RCTs of TCMIs combined with WM for ACI was conducted using electronic databases for studies dated from the start date of each database until February 2020. Stata 13.0 and ADDIS 1.16.7 software were used to plot and analyze the data. Sixty-six RCTs with a total of 5,989 patients involving 7 kinds of tonic TCMIs were included. Among TCMIs, Shenfu injection (SFI) + WM ranked first in terms of improving clinical efficacy and the activities of daily living (ADLs) rating and reducing interleukin-6 (IL-6) and tumor necrosis factor-*α* (TNF-*α*) levels. While Ciwujia injection (CI) + WM was the best choice for reducing neurological impairment and the high-cut viscosity of whole blood (HCV). Shenmai injection (SI) + WM had the greatest effects in terms of decreasing the levels of low-cut viscosity of whole blood (LCV), fibrinogen (FIB), and plasma viscosity (PV). Based on the cluster analysis of the clinical efficacy and the neurological impairment, CI + WM and Shenqifuzheng (SQI) + WM were the best options for treating ACI. With respect to adverse drug reactions (ADRs), 35 RCTs did not monitor ADRs during treatment. In conclusion, tonic TCMIs could assist WM in benefiting patients with ACI. However, due to the limitations of the current study, strict monitoring of ADRs and data from high-quality RCTs will be required in future to verify the advantage of TCMIs.

## 1. Introduction

Acute cerebral infarction (ACI) is caused by extensive or partial cerebrovascular occlusion or stenosis, resulting in an impaired blood supply to the brain, followed by cerebral ischemia and hypoxic necrosis, leading to brain damage and neurological deficit. In China, the annual growth rate of the incidence of stroke is 8.7%, ranking it as the primary cause of death among Chinese people, and exceeding the world average [1, 2]. ACI accounts for 70%–80% of all strokes [3]. ACI is a disease with high morbidity, disability, recurrence rate, and mortality, which not only seriously affects the quality of life of patients and their families but also brings heavy economic and psychological burdens to families and to society [4]. In the ultraearly stage of ACI, thrombolytic therapy, which rescues the ischemic penumbra and improves blood circulation in and around the infarct area to reduce or avoid secondary nerve damage, has become the consensus treatment [5, 6]. However, for patients beyond the time window of thrombolytic therapy or who cannot receive thrombolytic therapy for other reasons, the benefits provided by conventional treatment are limited [7, 8]; therefore, it is a practical clinical necessity to find an effective and safe adjuvant drug treatment for ACI.

Traditional Chinese medicine (TCM) has acquired centuries of experience at treating stroke, with a remarkable effect. The theory of TCM holds that “zheng qi deficiency” is one of the main factors involved in ACI; therefore, an approach of “supporting zheng qi and protecting the brain” allows the treatment of ACI to be broadened. Traditional Chinese medicine injections (TCMIs) are widely used to treat diseases [9–12] and possess characteristics of fast efficacy and high bioavailability [13–15]. In combination, TCMIs and Western medicine (WM) have become a supportive method for disease treatment in China [16–20]. Tonic TCMIs refer to TCMIs with tonic effects (such as tonifying qi and nourishing yin) as the main characteristic [21]. A large number of meta-analyses have shown that tonic TCMIs are able to treat ACI and have a significant effect on the condition [22–25]; however, the optimal treatment plan is still unclear because traditional pairwise comparison meta-analyses can only analyze and summarize a direct comparison of two interventions, which leads to certain limitations in evaluating the efficacy of multiple interventions. Network meta-analysis (NMA) is a development of traditional meta-analyses that simultaneously compares multiple different interventions with each other and analyzes the results of direct and indirect comparisons [26, 27]. In this study, the following 7 kinds of tonic TCMIs were selected as adjuvant therapies for ACI: Shengmai injection (SMI), Shenfu injection (SFI), Shenmai injection (SI), Huangqi injection (HQI), Ciwujia injection (CI), Dazhu Hongjingtian injection (DI), and Shenqifuzheng injection (SQI). NMA was used to systematically evaluate the efficacy of these tonic TCMIs in treating ACI and to rank them on different outcomes, providing data for evidence-based medicine that clinicians can use to choose more appropriate treatment options.

## 2. Materials and Methods

The abbreviations in the article are shown in Table S1. The PRISMA NMA checklist is shown in Table S2.

### 2.1. Inclusion Criteria

Randomized controlled trials (RCTs) that met the following four conditions were included. (1) Patients were diagnosed with ACI and had clear diagnostic criteria. (2) All patients in the RCT received conventional WM including dehydration, antiplatelet aggregation, correction of water and electrolyte disorders and acid–base imbalance, lipid-lowering, improvement of cerebral vascular circulation, cerebral nerve protection, and prevention of complications. The control group was given WM only or WM combined with a tonic TCMI, while the experimental group was given WM combined with a tonic TCMI. (3) As the primary outcome, the clinical efficacy was judged according to the decrease in the neurological deficit score: a reduction of 91%–100%, 46%–90%, 18%–45%, and ≤17% corresponds to “basic cure,” “notable progress,” “progress,” and “ineffectiveness” ratings, respectively [28]. The clinical efficacy rate was calculated using the following formula: (number of “basic cure” patients + number of “notable progress” patients + number of “progress” patients)/total number of patients × 100%. Neurological impairment was another primary outcome. The following were secondary outcomes: the activities of daily living (ADLs) rating, interleukin-6 (IL-6) level, tumor necrosis factor-*α* (TNF-*α*) level, the high-cut viscosity of whole blood (HCV), the low-cut viscosity of whole blood (LCV), fibrinogen (FIB) level, plasma viscosity (PV), and adverse reactions (ADRs). ADLs were assessed by the Barthel index [29]. (4) The study was designed solely as an RCT.

### 2.2. Exclusion Criteria

Studies that met the following requirements were excluded: (1) non-RCTs, (2) the full text of the study was not available, (3) incomplete or incorrect data, (4) RCTs did not meet the clinical efficacy valuation standard, (5) patients with serious complications, a bleeding tendency, severe liver and kidney dysfunction, or severe heart failure, and (6) interventions involving combination therapy with other TCMIs, or patients receiving acupuncture, surgery, or another physical therapy.

### 2.3. Search Strategy

All literature searches were conducted electronically. The searched databases comprised the following: the Chinese Biomedical Literature Database (CBM), the China National Knowledge Infrastructure Database (CNKI), the China Science and Technology Journal Database, the Wanfang Database, PubMed, Embase, and the Cochrane Library. All database searches were conducted on studies dating from the establishment of each database to February 19, 2020, with no language restrictions.  Table S3 shows the detailed terms used for the search.

### 2.4. Data Extraction

All of the RCTs found in the literature search were imported into NoteExpress software (Tongji University Library, Shanghai, China) and screened by two independent researchers (FZL and SSZ); if a disagreement was encountered, a third researcher resolved it (LQZ). Microsoft Excel 2016 (Microsoft, USA) was used to collect the information and data of the included RCTs as follows: (1) the name of the first author and the year of publication, (2) the number of patients and their gender and age, (3) the name, dose, and duration of the intervention, and (4) results regarding the following: clinical efficacy, neurological impairment, any ADRs, ADLs rating, TNF-*α* level, IL-6 level, FIB level, HCV, LCV, and PV.

### 2.5. Evaluation of Risk of Bias

The Cochrane risk of bias assessment tool was applied to assess the methodological quality of the included RCTs [30]; this tool assesses the following 7 items: (1) random sequence generation, (2) concealment of the distribution plan, (3) blinding of subjects and researchers, (4) blinding of the evaluators to the outcome, (5) the integrity of the final data, (6) selective reporting of research results, (7) other sources of bias. Each item includes three evaluation levels: low risk, unclear, and high risk. Two independent investigators (FZL and SSZ) assessed the quality of the included RCTs; if there was a disagreement, a third researcher (LDX) would resolve it.

### 2.6. Assessment of Transitivity Assumption

The validity of NMA is based on the transitivity assumption [31]. Transitivity indicates that intervention *C* is similar when it appears in the *C* versus *B* and *C* versus *A* trials [32]. An equivalent approach to evaluate transitivity is that trials which directly compare *C* with *A* and *C* with *B* would have similar effect modifiers distributions [33]. We assessed the transitivity assumption by comparing the distribution of the potential effect modifiers to ensure that they were on average balanced (which included age, sex, acute phase, and course). Control groups were evaluated for their similarity across treatment comparisons.

### 2.7. Statistical Analysis

Stata 13.0 software was used to plot a network graph of direct and indirect comparisons between different interventions under each outcome. Each node in the network graph represented an intervention, and the size of the node represented the number of RCTs for that intervention. A line between two nodes indicated that there was direct comparative evidence between the two interventions connected by it. The greater the number of comparisons, the thicker the connection. ADDIS 1.16.7 software was used to analyze the data. Dichotomous data were calculated using odds ratios (ORs), and continuous variables were calculated using mean differences (MDs). The 95% confidence interval (95% CI) was calculated for both ORs and MDs. The Markov chain Monte Carlo method was applied to fit the consistency model, which allowed it to be generated multiple times until convergence. The Bayesian model framework used the following parameters: number of chains = 4, number of turning iterations = 20,000, number of simulation iterations = 50,000, thinning interval = 10, number of inference samples = 10,000, and variance scaling factor = 2.5. The potential scale reduction factor (PSRF) was used to reflect the degree of convergence of the model. A PSRF value close to 1 indicated that the model had satisfied the convergence criterion and that the relevant results from the model could be used [34]. Combined results were considered statistically significant when the 95% CI of OR did not contain the value 1 or the 95% CI of MD did not contain the value 0 [35]. Given that the evidence network graphs were nonclosed loops, the consistency assumption between direct evidence and indirect evidence was not used. The surface under the cumulative ranking curve (SUCRA) was plotted to sort the treatments [36]. For the clinical efficacy and ADLs rating, a larger SUCRA value represented a better treatment option. In neurological impairment, levels of TNF-*α* and IL-6, and the HCV, LCV, FIB, and PV are inversely proportional to the efficacy; therefore, a lower SUCRA for a given intervention indicates that it is more effective at treating ACI. Based on the SUCRA, a cluster analysis was performed to compare the effects of tonic TCMIs on the clinical efficacy rate and on neurological impairment. The publication bias of the clinical efficacy data were tested using a funnel plot.

## 3. Results

### 3.1. Literature Search Results

**Figure 1:**
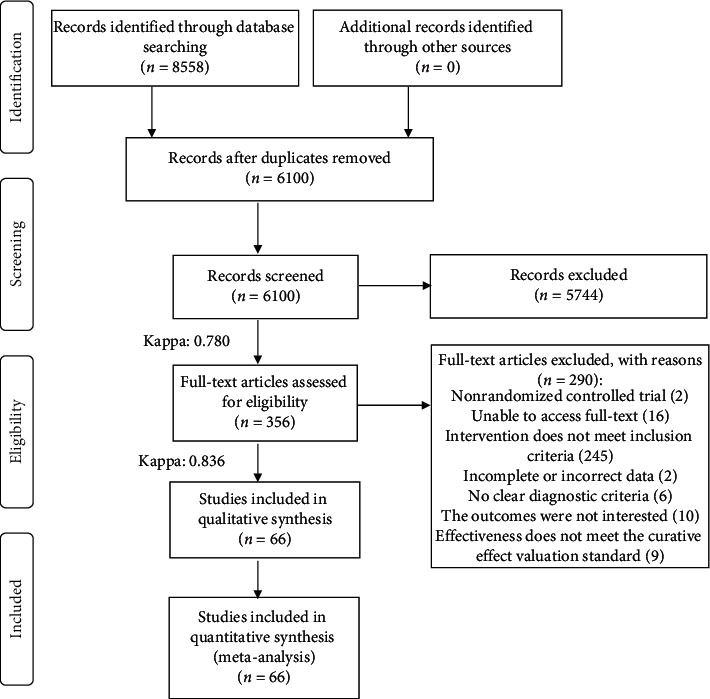
Flow chart and the kappa value for the literature screening process.

This study retrieved a total of 8,558 articles based on the literature search strategy employed. After deleting duplicate articles and filtering titles and abstracts, a total of 356 articles were obtained and further screened. There were 290 articles that did not meet the inclusion criteria for the following reasons: they were not RCTs; their results were irrelevant; the inclusion criteria were not met; the data were incomplete or incorrect; there was no clear diagnostic criteria; effectiveness did not meet the clinical efficacy valuation standard; or the full text could not be obtained. The final dataset of articles comprised 66 RCTs, which were included in the NMA. The process used for literature retrieval and screening is shown in Figure 1.

### 3.2. Study Characteristics

**Figure 2:**
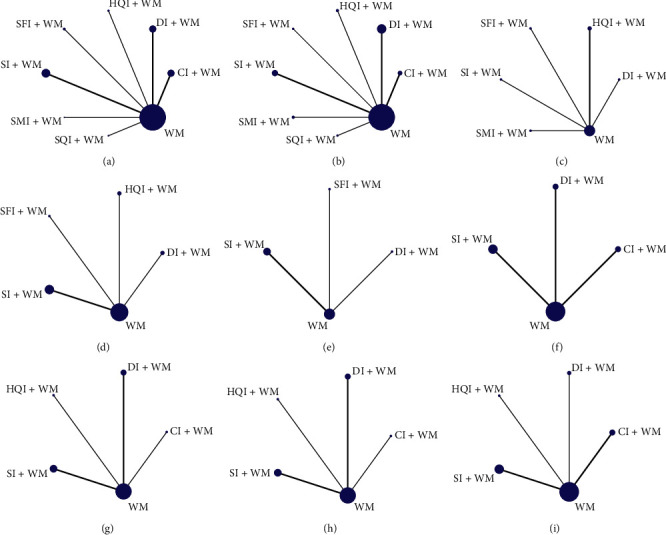
Network plots for the nine outcomes. (a) Clinical efficiency. (b) Neurological impairment. (c) ADLs rating. (d) TNF-*α* level. (e) IL-6 level. (f) FIB level. (g) HCV. (h) LCV. (i) PV. CI, Ciwujia injection; DI, Dazhu Hongjingtian injection; HQI, Huangqi injection; SFI, Shenfu injection; SI, Shenmai injection; SMI, Shengmai injection; SQI, Shenqifuzheng injection; WM, Western medicine.

A total of 66 RCTs involving 5,989 patients were included, all of whom were from mainland China, including 3,030 in the observation groups and 2,959 in the control groups. The minimum sample size was 30, and the maximum sample size was 200. These RCTs included 7 kinds of tonic TCMIs: SI (22 RCTs), DI (17 RCTs), CI (14 RCTs), HQI (4 RCTs), SFI (4 RCTs), SMI (3 RCTs), and SQI (2 RCTs). The tonic TCMIs were administered by intravenous drip in all cases.  Table S4 shows the characteristics of each study, and Figure 2 displays the network plots of each outcome.

### 3.3. Evaluation of  Transitivity Assumption

All patients had a diagnosis of ACI. The distribution with regard to age, sex, acute phase, and course was comparable between trials. All the control groups that received treatments were comparable, and their response rates were similar. Therefore, the transitivity assumption is tenable for our current dataset (Table S4).

### 3.4. Quality Assessment of Included Studies

**Figure 3:**
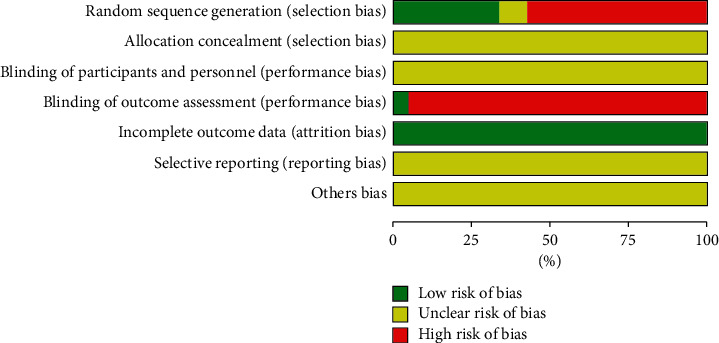
Risk of bias for all RCTs included in this study.

Regarding the generation of random sequences, 22 studies clearly stated the method used to generate random sequences and were considered “low risk;” 35 studies reported the method of generating random sequences as being “random” and 3 studies grouped patients according to their order of hospitalization, all of which were considered to be “high risk;” finally, 6 studies did not mention the generation of random sequences and were considered “unclear.” None of the studies reported information regarding the concealment of the distribution plan or the blinding of subjects and researchers, so they were all considered “unclear.” Two studies provided information on the blinding of outcome and were considered “low risk,” while the others were considered “high risk.” Selective reporting of outcome data and other biases were not confirmed in the studies and were considered “unclear.” Figure 3 shows the risk of bias for all the studies.

### 3.5. Outcomes of the NMA

#### 3.5.1. Clinical Efficacy

**Figure 4:**
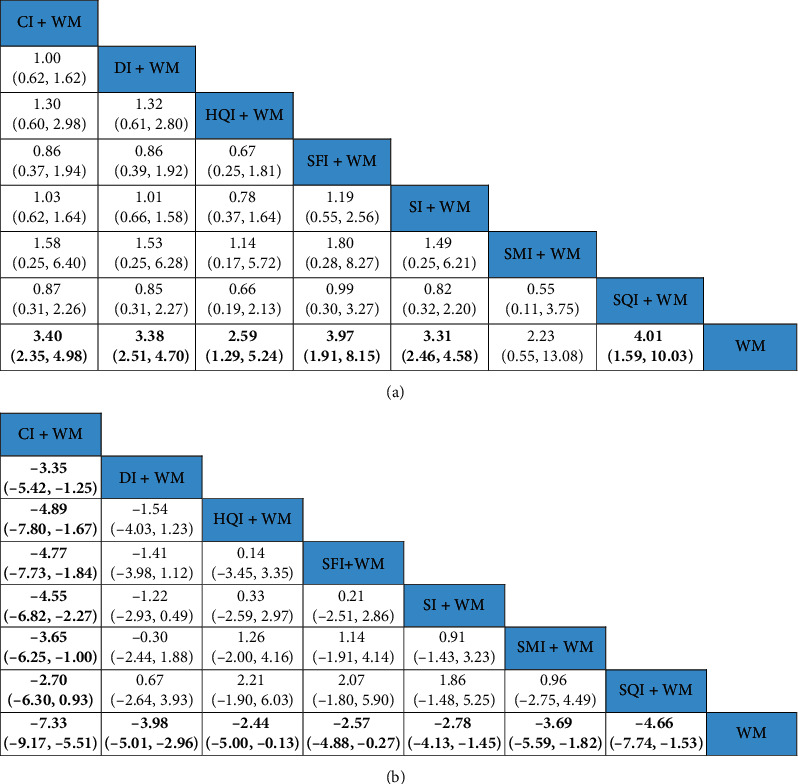
Results from the NMA showing the effect of each of the interventions. (a) ORs with 95% CIs of the clinical efficacy rate. (b) MDs with 95% CIs of the level of neurological impairment. The values in bold font represent statistically significant differences.

Fifty-two studies compared the clinical efficacies of a tonic TCMI combined with WM to WM alone. According to the comparisons from the NMA, the clinical efficacy of the following interventions were significantly stronger than that of WM: CI + WM (OR:3.40; 95% CI: 2.35, 4.98), DI + WM (OR: 3.38; 95% CI: 2.51, 4.70), HQI + WM (OR: 2.59; 95% CI: 1.29, 5.24), SFI + WM (OR: 3.97; 95% CI: 1.91, 8.15), SI + WM (OR: 3.31; 95% CI: 2.46, 4.58), and SQI + WM (OR: 4.01; 95% CI: 1.59, 10.03) (Figure 4(a)).

According to SUCRA plots, SFI + WM (74.88%) had the greatest likelihood of being the most effective treatment option in terms of improving the clinical efficacy of treatment for ACI patients, and the second was SQI (73%), as shown in Table 1.

#### 3.5.2. Neurological Impairment

The NMA of neurological impairment included 7 tonic TCMIs based on 35 RCTs on a total of 3,165 patients. As shown in Figure 4(b), the neurological impairment was obviously lower in the following interventions compared with that of WM alone: CI + WM (MD: −7.33; 95% CI: −9.17, −5.51), DI + WM (MD: −3.98; 95% CI: −5.01, −2.96), HQI + WM (MD: −2.44; 95% CI: −5.00, −0.13), SFI + WM (MD: −2.57; 95% CI: −4.88, −0.27), SI + WM (MD: −2.78; 95% CI: −4.13, −1.45), SMI + WM (MD: −3.69; 95% CI: −5.59, −1.82), and SQI + WM (MD: −4.66; 95% CI: −7.74, −1.53). The neurological impairment for CI + WM was lower than those for the following interventions: DI + WM (MD: −3.35; 95% CI: −5.42, −1.25), HQI + WM (MD: −4.89; 95% CI: −7.80, −1.67), SFI + WM (MD: −4.77; 95% CI: −7.73, −1.84), SI + WM (MD: −4.55; 95% CI: −6.82, −2.27), and SMI + WM (MD: −3.65; 95% CI: −6.25, −1.00). As shown in Table 1, the SUCRA showed that CI + WM (13.38%) had the highest likelihood of being the best treatment for reducing neurological impairment in ACI patients.

#### 3.5.3. ADLs Rating

**Figure 5:**
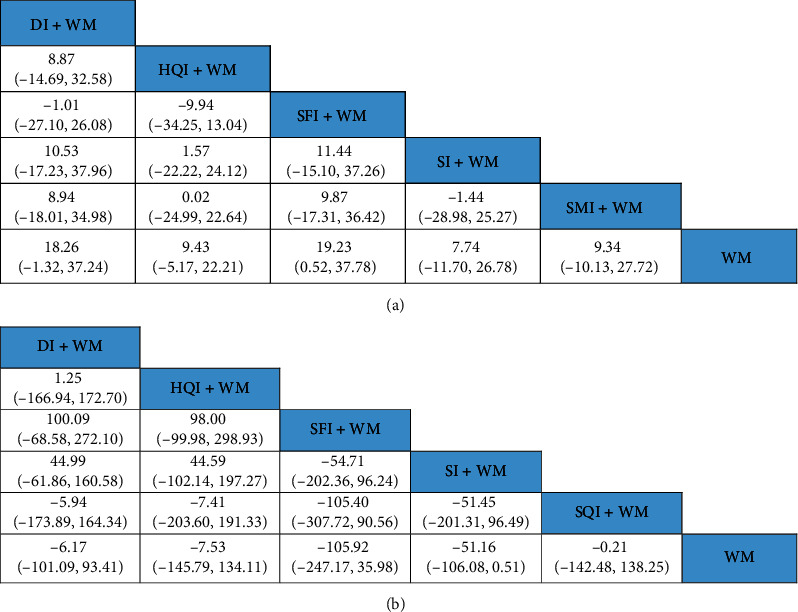
NMA comprising the effects of all treatment interventions. (a) ORs with 95% CIs for ADLs rating. (b) MDs with 95% CIs of the TNF-*α* level. The values in bold font represent statistically significant differences.

The NMA of ADLs rating included 5 kinds of tonic TCMIs based on 6 RCTs with 484 patients. As shown in Figure 5(a), there were no significant differences among the 5 tonic TCMIs. Based on the SUCRA, SFI + WM (86.67%) ranked first (Table 1).

#### 3.5.4. TNF-*α* Level

The NMA of the TNF-*α* level included 5 kinds of tonic TCMIs based on 12 RCTs with 1,102 patients.  Figure 5(b) shows that there were no significant differences between each comparison in the TNF-*α* level. The SUCRA indicated that SFI + WM (26.83%) had the strongest effect on reducing TNF-*α* levels (Table 1).

#### 3.5.5. IL-6 Level

**Figure 6:**
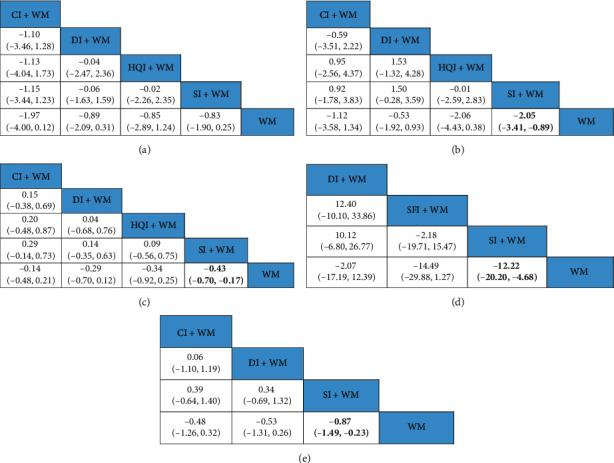
NMA comprising the effects of all treatment interventions. (a) MDs with 95% CIs of the HCV. (b) MDs with 95% CIs of the LCV. (c) MDs with 95% CIs of the PV. (d) MDs with 95% CIs of the IL-6 level. (e) MDs with 95% CIs of the FIB level. The values in bold font represent statistically significant differences.

The NMA of the IL-6 level included 3 TCMIs based on 6 RCTs with 570 patients. As shown in Figure 6(d), the IL-6 level for SI + WM (MD: −12.22; 95% CI: −20.20, −4.68) was lower than that for WM. The rank probability showed that SFI (36.75%) had the strongest effect on reducing IL-6 levels (Table 1).

#### 3.5.6. FIB Level

The NMA of FIB levels included 3 TCMIs based on 12 RCTs with 1,075 patients. As shown in Figure 6(e), FIB levels for SI + WM (MD: −0.87; 95% CI: −1.49, −0.23) were lower than that for WM. The SUCRA showed that SI + WM (35.25%) was most likely to be the best treatment for reducing FIB levels (Table 1).

#### 3.5.7. HCV and LCV Levels

The NMA of HCV and LCV levels included 4 TCMIs based on 9 RCTs with 884 patients.  Figure 6(a) shows that there were no significant differences between each comparison in HCV levels. LCV levels were lower for SI + WM (MD: −2.05; 95% CI: −3.41, −0.89) relative to that for WM (Figure 6(b)). According to the SUCRA, CI + WM (29.8%) was better than the other treatments at reducing HCV levels, while SI + WM (34.2%) had the highest likelihood of being the best treatment for decreasing LCV levels (Table 1).

#### 3.5.8. PV Level

The NMA of PV levels included 4 TCMIs based on 11 RCTs with 950 patients. As shown in Figure 6(c), the PV levels for SI + WM (MD: −0.43; 95% CI: −0.70, −0.17) were lower than that of WM. The SUCRA suggested that SI + WM (34.6%) was the best combination therapy for decreasing PV levels (Table 1).

#### 3.5.9. ADRs

Of the 66 RCTs included in this study, 31 RCTs (46.97%) reported ADRs during treatment, of which 11 RCTs reported ADRs in detail; 2 RCTs only reported the number of ADRs in different groups, but did not report specific symptoms; and 18 RCTs reported no obvious ADRs. For the SI treatment intervention, 8 RCTs reported ADRs, including gastrointestinal symptoms (8 cases), abnormal skin (4 cases), oppression in the chest (3 cases), flushing (3 cases), bleeding (1 case), and elevated transaminases (1 case). Two RCTs reported ADRs following SFI treatment, including gastrointestinal symptoms (3 cases), itching of the skin (4 cases), increased transaminases (1 case), hematuria (1 case), increased blood creatinine (1 case), and abnormal liver and spleen function (2 cases). One RCT reported that treatment with CI led to epistaxis (2 cases) and gum bleeding (1 case). The control groups also had the above ADRs, but there was no significant difference compared with the treatment groups. A total of 35 RCTs (53.03%) did not monitor ADRs during treatment.

#### 3.5.10. Publication Bias

**Figure 7:**
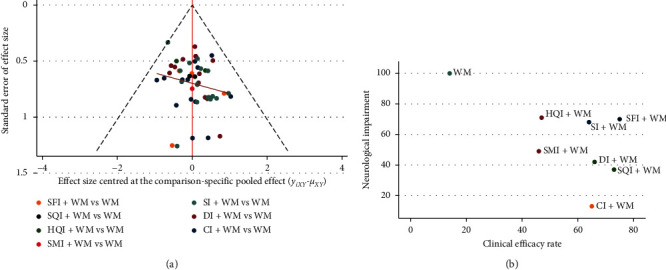
Publication bias and cluster analysis. (a) Publication bias for the analysis of clinical efficacy. (b) Cluster analysis for the neurological impairment and the clinical efficacy rate. Treatments located in the lower right corner are the best treatments with respect to the neurological impairment and the clinical efficacy rate.

Based on the clinical efficacy values obtained, a funnel plot was used to assess publication bias.  Figure 7(a) shows differently colored points representing comparisons among the different interventions. The adjusted auxiliary line showed an angle with the midline, which suggests that this study had a small publication bias.

#### 3.5.11. Cluster Analysis

This study conducted a cluster analysis of the neurological impairment and the clinical efficacy rate; the results are shown in Figure 7(b). Based on comprehensive analysis of these clusters, CI + WM and SQI + WM were determined to be the most beneficial in terms of their effects on the clinical efficacy rate and neurological impairment.

## 4. Discussion

In China, ACI is the leading cause of death and the main cause of disability for residents and has become a public health problem [37, 38]. According to TCM theory, “zheng qi deficiency” is the main cause of ACI, while tonic TCMIs are important for tonifying deficiency. As an adjuvant treatment for ACI, TCMIs have been shown to exert an obvious effect on the condition. In this study, we conducted an NMA of 7 types of tonic TCMIs and combined the outcomes to determine which injection is the best choice for clinical treatment and to provide a reference for clinicians to treat ACI.

The NMA included 66 RCTs involving 5,989 patients and evaluated the clinical efficacy, neurological impairment, ADLs rating, levels of inflammatory factors (TNF-*α* and IL-6), and hemorheological changes (FIB level, and HCV, LCV, and PV) of interventions comprising a tonic TCMI combined with WM. The results showed that compared with WM alone, tonic TCMIs combined with WM yielded obvious therapeutic benefits for patients with ACI. The results suggest that SFI + WM is the optimal treatment plan for increasing clinical efficacy, while CI + WM is most likely to be the best treatment for ameliorating neurological impairment. The effect of SFI according to TCM is to reinforce qi and restore yang, and the effective ingredients in SFI have been determined to be various ginsenosides and aconitine [39]. Pairwise comparison of meta-analyses showed that SFI clearly improves the clinical efficacy of treating cerebral infarction [22]. SQI is made by the extraction and separation of *Codonopsis pilosula* and *Astragalus*, with the main compounds in these tonics including flavonoids, saponins, and lignans, whose efficacy in TCM is via the invigoration of qi and the promotion of blood circulation [40]. SQI has an obvious protective effect on the heart, brain, and kidney [41]. A meta-analysis showed that SQI clearly improves the clinical efficacy of treating cerebral infarction [42]. Meanwhile, CI is extensively applied in the treatment of coronary heart disease and stroke [43, 44]. The function of CI in TCM is to reinforce the qi strength of the spleen, nourish the liver and kidney, and to activate the blood. Modern pharmacological studies have shown that CI contains a variety of saponins, polysaccharides, and flavonoids, which function to dilate blood vessels, lower blood pressure, induce antiplatelet aggregation, ameliorate hemorheological properties, improve microcirculation, and increase blood supply to the brain [45]. An in vitro study showed that CI was able to protect PC12 cells, a cell model that mimics neurons, from neurotoxin-induced damage [46].

The inflammatory response during ACI has been a focus of research in recent years. Inflammatory factors participate in the ischemic cascade reaction, which further aggravates the symptoms of cerebral ischemia and the degree of brain damage [47]. TNF-*α* and IL-6 are the main inflammatory factors involved in brain tissue damage; they are expressed at high levels in the early stage of ACI and are related to the infarct volume, neurological deficit, and prognosis after stroke [48, 49]. In the present study, the SUCRA showed that SFI was the most effective treatment for reducing TNF-*α* and IL-6 levels. Animal experiments have shown that SFI significantly inhibits the levels of TNF-*α* and IL-6 and exerts an obvious anti-inflammatory effect [50–52]. Clinical studies have also determined that SFI protects the body from damage by inhibiting inflammatory factors [53, 54]. The SUCRA in the present study also showed that SFI ranked the highest in terms of improving ADLs rating. The ginsenosides in SFI contribute additional effects toward inhibiting apoptosis, eliminating free radicals, reducing oxidative damage, and inhibiting calcium overload; therefore, SFI is able to reduce nerve damage, thereby improving the ADLs of patients [55].

An increase in the levels of hemorheological indicators is one critical risk factor for stroke [56, 57]. Abnormalities in hemorheology have an important impact on the formation of blood clots, and whole blood viscosity, PV, and FIB are all important indicators reflecting hemorheology. An increase in their levels indicates that the ability of erythrocytes to aggregate is increased, and their deformability is decreased, which directly promotes the formation of a thrombus [58, 59]. One study showed that the higher the level of hemorheological indicators, the worse the improvement of neurological deficits in patients with stroke [60]. In the treatment of ACI, reducing blood viscosity, blood hypercoagulability, and FIB levels, antiplatelet aggregation, and improving cerebral blood circulation have important clinical significance. The results of the current study suggest that SI + WM is the best treatment for reducing the FIB level, LCV, and PV. The effect of SI according to TCM is to reinforce the qi and nourish yin. The main components of SI include ginsenosides and ophiopogon saponin [61, 62]. The ginsenoside Rd has clearly been shown to reduce hemorheological indices after ACI [63].

In addition to the clinical benefits of a treatment, ADRs also need to be considered with respect to clinical medication. The current study found that only 13 RCTs reported clear ADRs, while 35 RCTs did not report them; therefore, our research cannot draw any firm conclusions regarding ADRs. The ADRs in response to TCM injections are caused by the injection itself, the constitution of the patient, and improper use of the drugs [64]. Studies have shown that abnormal skin, allergic reactions, gastrointestinal symptoms, and bleeding were the most common ADRs for TCMIs [65]. Our existing evidence showed that there is no significant difference in ADRs between TCMIs and WM. Therefore, future RCTs need to monitor the ADRs of TCMIs and WM strictly, and TCMIs must be administered in accordance with the specifications and under the guidance of clinicians.

In this study, Bayesian NMA was used to evaluate the efficacy of tonic TCMIs in the treatment of ACI and to provide a basis for clinicians to choose appropriate treatment options. We established strict inclusion and exclusion criteria to reduce the clinical heterogeneity of interventions and disease conditions between included RCTs. Although clinical heterogeneity cannot be eliminated completely, it can be reduced in this way. After ACI, the recovery of nerve function and the changes of inflammatory factors and hemorheology levels are crucial to the prognosis of patients. This Bayesian NMA used clinical efficacy, neurological impairment, and ADLs to assess the overall rehabilitation status of ACI patients, TNF-*α* and IL-6 to reflect the levels of inflammation to determine the patient's condition, and hemorheology indicators to identify the blood characteristics.

Our current research had three advantages. First, this NMA is the first to compare the therapeutic effects of tonic TCMIs on ACI, based on a comprehensive search of the literature. Second, strict inclusion and exclusion criteria were established. Third, in addition to analyzing clinical efficacy, neurological impairment, and ADLs rating, we also analyzed changes in inflammatory factors and hemorheological indices, which have some importance in guiding the treatment of ACI. However, our research also had limitations. Only 33.33% (22/66) of the RCTs analyzed clearly described the method of random sequence generation used. None of the studies provided information regarding the concealment of the distribution plan or of the blinding of subjects and researchers; therefore, the methodological quality of the included RCTs was not high. All the patients recruited in the RCTs were from China, with recruitment of patients with ACI from other countries lacking; hence, the results of our research are not universally applicable. With regard to the outcomes of the ADLs rating and IL-6 level, fewer RCTs were included, which may have weakened the strength of evidence supporting the results. Moreover, most of the results from the included RCTs were based on comparing combined treatment with a tonic TCMI and WM to WM alone and lacked direct comparison among TCMIs. In future, RCTs of TCMIs for treating ACI should be improved.

## 5. Conclusions

Tonic TCMIs may be able to assist WM in benefiting patients with ACI. Based on the neurological impairment and the clinical efficacy rate, CI + WM and SQI + WM were found to be the best options for treating ACI. Considering the ADLs rating, TNF-*α* level, and IL-6 level, SFI + WM was superior to all of the other treatments. SI + WM had the greatest beneficial effects over other treatments in terms of decreasing levels of FIB, LCV, and PV. However, due to the low methodological quality related to the enrolled RCTs in this study, high-quality multicenter RCTs are needed to further verify our conclusions.

## Figures and Tables

**Table 1:** SUCRA values (%) of each therapeutic intervention for the listed outcomes.

Treatments	Outcomes								
	A	B	C	D	E	F	G	H	I
CI + WM	65.25	**13.38**	—	—	—	61.5	**29.8**	60.2	72.2
DI + WM	65.5	42.25	83.33	68.67	79	57.75	57	74.8	52.2
HQI + WM	47	70.38	53.5	67	—	—	59.6	37.3	49
SFI + WM	**74.88**	70.13	**86.67**	**26.83**	**36.75**	—	—	—	—
SI + WM	63.75	67.63	48.33	41.5	42.5	**35.25**	59	**34.2**	**34.6**
SMI + WM	46.25	49	54.17	—	—	—	—	—	—
SQI + WM	73	37.13	—	69.83	—	—	—	—	—
WM	14.13	99.5	22.17	75.33	90.75	94.75	93.8	92.8	93

*Note*. *A*, clinical efficacy; *B*, neurological impairment; *C*, ADLs rating; *D*, TNF-*α*; *E*, IL-6; F, FIB; *G*, HCV; *H*, LCV; *I*, PV. CI, Ciwujia injection; DI, Dazhu Hongjingtian injection; HQI, Huangqi injection; SFI, Shenfu injection; SI, Shenmai injection; SMI, Shengmai injection; SQI, Shenqifuzheng injection; WM, Western medicine. The values in bold font represent the best therapeutic intervention for each outcome

## Data Availability

The data used to support the findings of this study are all from published studies and have been cited in this study.

## Supplementary Materials

Supplementary Materials Supplementary 1. Table S1: Abbreviations. Supplementary 2. Table S2: PRISMA NMA checklist. Supplementary 3. Table S3: strategy for searching PubMed; Table S4: characteristics of included RCTs.
