# Supplementary material for: Clinical Efficacy of Tonic Traditional Chinese Medicine Injection on Acute Cerebral Infarction: A Bayesian Network Meta-Analysis
Source: Evid Based Complement Alternat Med. 2020 Nov 23;2020:8318792. doi: 10.1155/2020/8318792 (PMC7704142; doi:10.1155/2020/8318792)
Supplement: Supplementary Materials — Supplementary 1. Table S1: Abbreviations. Supplementary 2. Table S2: PRISMA NMA checklist. Supplementary 3. Table S3: strategy for searching PubMed; Table S4: characteristics of included RCTs. [file 8318792.f1.zip › 8318792.f1/Supplementary 1 (1).docx]

**Supplementary 1**

Table S1 Abbreviations.

| Abbreviations | Full name |
| --- | --- |
| ACI | Acute cerebral infarction |
| WM | Western medicine |
| TCM | Traditional Chinese medicine |
| TCMI | Traditional Chinese medicine injection |
| RCT | Randomized controlled trial |
| NMA | Network meta-analysis |
| ADL | Activity of daily living |
| TNF-α | Tumor necrosis factor-α |
| IL-6 | Interleukin-6 |
| HCV | High-cut viscosity of whole blood |
| LCV | Low-cut viscosity of whole blood |
| FIB | Fibrinogen |
| PV | Plasma viscosity |
| ADR | Adverse drug reaction |
| SMI | Shengmai injection |
| SFI | Shenfu injection |
| SI | Shenmai injection |
| HQI | Huangqi injection |
| CI | Ciwujia injection |
| DI | Dazhu hongjingtian injection |
| SQI | Shenqifuzheng injection |
| OR | Odds ratio |
| MD | Mean difference |
| 95% CI | 95% confidence interval |
| SUCRA | Surface under the cumulative ranking curve |
