# Supplementary material for: Clinical Efficacy of Tonic Traditional Chinese Medicine Injection on Acute Cerebral Infarction: A Bayesian Network Meta-Analysis
Source: Evid Based Complement Alternat Med. 2020 Nov 23;2020:8318792. doi: 10.1155/2020/8318792 (PMC7704142; doi:10.1155/2020/8318792)
Supplement: Supplementary Materials — Supplementary 1. Table S1: Abbreviations. Supplementary 2. Table S2: PRISMA NMA checklist. Supplementary 3. Table S3: strategy for searching PubMed; Table S4: characteristics of included RCTs. [file 8318792.f1.zip › 8318792.f1/Supplementary 3 (1).docx]

**Supplementary 3**

Table S3 Strategy for searching PubMed.

| Search | Query | Items found |
| --- | --- | --- |
| #1 | Stroke[Title/Abstract] | [239369](https://www.ncbi.nlm.nih.gov/pubmed/?cmd=HistorySearch&querykey=1) |
| #2 | Apoplexy[Title/Abstract] | [3078](https://www.ncbi.nlm.nih.gov/pubmed/?cmd=HistorySearch&querykey=2) |
| #3 | Cerebral Stroke[Title/Abstract] | [1403](https://www.ncbi.nlm.nih.gov/pubmed/?cmd=HistorySearch&querykey=3) |
| #4 | Acute cerebral infarction[Title/Abstract] | 1236 |
| #5 | ACI[Title/Abstract] | 3166 |
| #6 | Acute ischemic stroke[Title/Abstract] | 14129 |
| #7 | AIS[Title/Abstract] | 11178 |
| #8 | Cerebrovascular Apoplexy[Title/Abstract] | 4 |
| #9 | Cerebrovascular Stroke[Title/Abstract] | 310 |
| #10 | Stroke, Acute[Title/Abstract] | 9 |
| #11 | Brain infarction[Title/Abstract] | 2404 |
| #12 | Anterior Circulation Brain Infarction[Title/Abstract] | 1 |
| #13 | Venous Infarction, Brain[Title/Abstract] | 819 |
| #14 | Infarction, Venous Brain[Title/Abstract] | 819 |
| #15 | Venous Brain Infarction[Title/Abstract] | 77 |
| #16 | Anterior Cerebral Circulation Infarction[Title/Abstract] | 3 |
| #17 | Posterior Circulation Infarction, Brain[Title/Abstract] | 315 |
| #18 | #1 OR #2 OR #3 OR #4 OR #5 OR #6 OR #7 OR #8 OR #9 OR #10 OR #11 OR #12 OR #13 OR #14 OR #15 OR #16 OR #17 | [256448](https://www.ncbi.nlm.nih.gov/pubmed/?cmd=HistorySearch&querykey=18) |

Table S3 Search strategy of PubMed (Continued).

| Search | Query | Items found |
| --- | --- | --- |
| #19 | Shenqifuzheng injection[Title/Abstract] | 5 |
| #20 | Shenqifuzheng[Title/Abstract] | 10 |
| #21 | Huangqi[Title/Abstract] | 270 |
| #22 | Huangqi injection[Title/Abstract] | 25 |
| #23 | Astragalus[Title/Abstract] | 2805 |
| #24 | Astragalus injection[Title/Abstract] | 63 |
| #25 | Shenmai[Title/Abstract] | 212 |
| #26 | Shenmai injection[Title/Abstract] | 151 |
| #27 | Shenfu[Title/Abstract] | 183 |
| #28 | Shenfu injection[Title/Abstract] | 138 |
| #29 | Shengmai[Title/Abstract] | 171 |
| #30 | Shengmai Injection[Title/Abstract] | 68 |
| #31 | Ciwujia injection[Title/Abstract] | 8 |
| #32 | Ciwujia[Title/Abstract] | 22 |
| #33 | Acanthopanax Senticosus injection[Title/Abstract] | 4 |
| #34 | Acanthopanax Senticosus[Title/Abstract] | 203 |
| #35 | Hongjingtian injection[Title/Abstract] | 3 |
| #36 | Hongjingtian[Title/Abstract] | 15 |
| #37 | Rhodiola injection[Title/Abstract] | 45 |
| #38 | Rhodiola[Title] | 517 |
| #39 | Dazhu hongjingtian injection[Title/Abstract] | 0 |
| #40 | Dazhu hongjingtian[Title/Abstract] | 5 |

Table S3 Search strategy of PubMed (Continued).

| Search | Query | Items found |
| --- | --- | --- |
| #41 | #19 OR #20 OR #21 OR #22 OR #23 OR #24 OR #25 OR #26 OR #27 OR #28 OR #29 OR #30 OR #31 OR #32 OR #33 OR #34 OR #35 OR #36 OR #37 OR #38 OR #39 OR #40 | 4302 |
| #42 | #18 AND #41 | 61 |
| #43 | Randomized controlled trial[Title/Abstract] | [70252](https://www.ncbi.nlm.nih.gov/pubmed/?cmd=HistorySearch&querykey=47) |
| #44 | RCT[Title/Abstract] | [22473](https://www.ncbi.nlm.nih.gov/pubmed/?cmd=HistorySearch&querykey=48) |
| #45 | #43 OR #44 | [86883](https://www.ncbi.nlm.nih.gov/pubmed/?cmd=HistorySearch&querykey=49) |
| #46 | #42 AND #45 | 2 |

Table S4 Characteristics of included RCTs

| Stu­dy ID | N (E/C) | Gender  (M/F) | Age  (years, mean±SD) | Experimental  group | Control  group | Acute  phase | Course | Outcomes |
| --- | --- | --- | --- | --- | --- | --- | --- | --- |
| Cao CY 2016 [1] | 50 (25/25) | 35/15 | 47-85  (65.4±3.5) | SI 50ml + WM | WM | <24h | 14d | ①⑩ |
| Chen HJ 2019 [2] | 100 (50/50) | 58/42 | 55-68  E: (63.01±8.19)  C: (62.85±8.18) | DI 10ml + WM | WM | <48h | 14d | ①⑥⑦⑧ |
| Chen HY 2018 [3] | 84 (42/42) | 44/40 | 48-77  E: (59.84±5.47)  C: (59.06±5.13) | DI 10ml + WM | WM | <24h | 14d | ①②④⑤ |
| Chen JB 2019 [4] | 200 (100/100) | 104/96 | 35-80  (52.6±4.8) | CI 400mg + WM | WM | 2-14h | 20d | ①②⑩ |
| Chen S 2014 [5] | 131 (64/67) | 67/64 | 48-77  E: (63.6)  C: (63.1) | DI 10ml + WM | WM | <48h | 10d | ①② |
| Chen ZM 2018 [6] | 94 (47/47) | 55/39 | 70-89  E: (78.47±5.62)  C: (78.91±6.13) | SI 60ml + WM | WM | 3-46h | 14d | ①④ |
| Cheng PR 2015 [7] | 86 (44/42) | 54/32 | 41-78 | SMI / +WM | WM | <48h | 20d | ② |
| Cui H 2013 [8] | 135 (68/67) | / | / | DI 10ml + WM | WM | <14d | 30d | ①⑩ |
| Du XL 2004 [9] | 60 (30/30) | 33/27 | 50-80  (67.6±6.6) | CI 60ml + WM | WM | <3d | 14d | ②⑥ |
| Fan B 2009 [10] | 94 (49/45) | 46/48 | 40-81  E: (57±20.7)  C: (59±17.6) | HQI 40ml + WM | WM | 6-72h | 14d | ①②③⑦⑧⑨ |
| Fan RM 2019 [11] | 96 (48/48) | 47/49 | 52-78  E: 69.6  C: 67.9 | DI 10ml + WM | WM | 3h-2d | 28d | ②⑩ |
| Fei XD 2017 [12] | 30 (15/15) | 16/14 | 44-82  E: 60.3  C: 63.4 | DI 10ml + WM | WM | <14d | 9d | ①⑩ |
| Fu KQ 2010 [13] | 162 (84/78) | 119/43 | / | SI 50ml + WM | WM | / | 14d | ① |
| Han M 2018 [14] | 108 (54/54) | 65/43 | 45-82  E: (65.18±6.72)  C: (64.57±6.85) | SI 60-100ml + WM | WM | 6-24h | 28d | ④⑤ |
| He T 2017 [15] | 80 (40/40) | 45/35 | 45-76  E: (61.1±2.9)  C: (60.3±2.3) | DI 10ml + WM | WM | <24h | 14d | ② |
| He ZC 2010 [16] | 60 (30/30) | 40/20 | 50-75  E: (61.3±6.7)  C: (62.7±7.3) | HQI 30ml + WM | WM | / | 14d | ①②④ |
| Hu AM 2004 [17] | 60 (30/30) | 34/26 | 29-84  E: 63.4  C: 64.9 | CI 60-80ml + WM | WM | 1-3d | 14d | ①② |
| Hu M 2018 [18] | 114 (57/57) | 54/60 | 47-76  E: (60.11±10.24)  C: (61.24±10.43) | SI 40ml + WM | WM | <24h | 14d | ①②⑩ |
| Huang X 2009 [19] | 86 (48/38) | 51/35 | 49-73  (63.4±5.6) | SQI 250ml + WM | WM | <72h | 14d | ①②⑩ |
| Jiang KJ 2003 [20] | 106 (52/54) | 74/32 | 50-75  E: (63±6.7)  C: (65.7±7.39) | CI 60ml + WM | WM | <3d | 14d | ①② |
| Li AH 2007 [21] | 80 (40/40) | 51/29 | 42-75  (58.6) | SI 50ml + WM | WM | <72h | 14d | ①⑥⑦⑧⑨⑩ |
| Li DX 2008 [22] | 93 (52/41) | 69/24 | 43-81  E: (61±18)  C: (62±19) | CI 30ml + WM | WM | <72h | 10d | ①⑥⑩ |
| Li QY 1997 [23] | 120 (60/60) | 77/43 | / | HQI 20ml + WM | WM | 3h-7d | 14d | ①⑩ |
| Li RH 2002 [24] | 68 (32/36) | 47/21 | 40-78  E: 63  C: 65 | SI 30-50ml + WM | WM | <3d | 14d | ⑩ |
| Li XP 2019 [25] | 80 (40/40) | 46/34 | 52-74  (65.2±6.4) | SI 50ml + WM | WM | <48h | 14d | ①②④⑩ |
| Li YY 2019 [26] | 140 (70/70) | 73/67 | 63-84  E: (73.5±3.4)  C: (73.1±3.9) | SI 50ml + WM | WM | 6-48h | 14d | ①②⑩ |
| Liang XL 2018 [27] | 80 (40/40) | 52/28 | 18-80  E: (58.8±14.87)  C: (56.9±15.24) | SI 50ml + WM | WM | <5d | 21d | ①②⑩ |
| Lin QK 2013 [28] | 78 (39/39) | 41/37 | 42-75  (59.72±7.53) | SMI 40ml + WM | WM | 15-124min | 28d | ①②③ |
| Liu D 2017 [29] | 120 (60/60) | 74/46 | 42-78  E: (60.34±2.31)  C: (61.13±2.25) | SFI 50ml + WM | WM | 7-31h | 14d | ①②③④⑤⑩ |
| Liu SJ 2016 [30] | 64 (32/32) | 40/24 | 32-76 | DI 10ml + WM | WM | / | 14d | ①② |
| Long ZM 2000 [31] | 60 (30/30) | 43/17 | 48-72  E: (60.5±8.7)  C: (58.5±7.3) | CI 80ml + WM | WM | / | 14d | ①⑨ |
| Lu R 2013 [32] | 64 (32/32) | 35/29 | 48-70  E: (57.5±8.2)  C: (58.4±7.8) | SI 40m l + WM | WM | <24h | 14d | ①②③④⑤ |
| Mei HX 2013 [33] | 120 (60/60) | 72/48 | 45-75  E: 61.80  C: 62.81 | DI 10ml + WM | WM | <24h | 60d | ①② |
| Peng CJ 2006 [34] | 100 (50/50) | 59/41 | 40-77 | CI 250ml + WM | WM | 6h-3d | 14d | ① |
| Qin PS 2001 [35] | 86 (43/43) | 48/38 | 36-72  E: (63.54±7.63)  C: (62.34±8.56) | CI 60ml + WM | WM | <24h | 14d | ①⑦⑧⑨ |
| Sha LL 2013 [36] | 160 (80/80) | 99/61 | 52-70  E: 59.4  C: 62.6 | DI 10ml + WM | WM | <24h | 14d | ① |
| Tang YG 2011 [37] | 65 (35/30) | 33/32 | 51-79  (66.5) | SI 60ml + WM | WM | <48h | 14d | ①②⑩ |
| Wang HZ 2013 [38] | 200 (100/100) | 107/93 | 43-78  (61.6±9.2) | SI 30ml + WM | WM | <72h | 20d | ① |
| Wang JM 2015 [39] | 105 (54/51) | 59/46 | 45-67  (60.4±5.1) | DI 10ml + WM | WM | <24h | 14d | ①② |
| Wang JX 2014 [40] | 56 (28/28) | 34/22 | 45-72  E: (62.5±8.2)  C: (59.6±8.5) | SI 50ml + WM | WM | <24h | 14d | ①② |
| Wang L 2014 [41] | 120 (65/55) | 70/50 | 36-81  E: (56.8±6.9)  C: (57.3±7.2) | SI 40ml + WM | WM | 0.5-13h | 28d | ④⑤⑥⑦⑧⑨ |
| Wang Q 2013 [42] | 60 (30/30) | 36/24 | 38-75  (55.3) | SI 60ml + WM | WM | <48h | 14d | ④⑤⑥⑦⑧⑨⑩ |
| Wang XP 2019 [43] | 92 (46/46) | 42/50 | 53-76  E: (64.5±5.0)  C: (65.2±5.1) | DI 30mg + WM | WM | 2-23h | 28d | ②④⑩ |
| Wang YP 2006 [44] | 60 (30/30) | 34/26 | 55-79 | CI 80ml + WM | WM | 8-48h | 15d | ① |
| Wang ZY 2005 [45] | 64 (32/32) | 38/26 | E: (63.21±11.25)  C: (62.41±11.68) | CI 60ml + WM | WM | <7d | 14d | ① |
| Wei YL 2017 [46] | 60 (30/30) | 35/25 | 51-79 | DI 10ml + WM | WM | <72h | 28d | ①②⑩ |
| Wu PH 2015 [47] | 40 (20/20) | 24/16 | (61.45±8.9) | SMI 60ml + WM | WM | / | 14d | ② |
| Xia LL 2012 [48] | 135 (68/67) | 69/66 | 44-82  E: 63.5  C: 62.3 | DI 10ml + WM | WM | <14d | 14d | ①⑥⑦⑧⑨⑩ |
| Xia LL 2013 [49] | 149 (75/74) | 75/74 | 41-83  E: (68±3.2)  C: (69±2.8) | DI 10ml + WM | WM | <14d | 28d | ①②⑥⑦⑧⑨⑩ |
| Xiao CY 2007 [50] | 62 (37/25) | 37/25 | 42-72  E: 64.5  C: 63.3 | CI 250ml + WM | WM | / | 28d | ① |
| Xu HB 2001 [51] | 60 (30/30) | 40/20 | 45-67  E: 56.4  C: 62.2 | SI 40ml + WM | WM | <48h | 10d | ①⑩ |
| Xu LB 2010 [52] | 105 (55/50) | 84/21 | 37-85  E: 62.3  C: 63.4 | SFI 50ml + WM | WM | 6-72h | 14d | ①⑩ |
| Xu PW 2003 [53] | 112 (56/56) | 63/49 | 49-73  E: 61  C: 63 | CI 60ml + WM | WM | <48h | 14d | ①⑥⑩ |
| Xue F 2007 [54] | 60 (30/30) | 41/19 | 40-70  (57.6) | SI 50ml + WM | WM | <72h | 14d | ①⑥⑦⑧⑨⑩ |
| Yang JS 2016 [55] | 96 (48/48) | 61/35 | 45-82  E: (66.8±2.4)  C: (65.4±2.3) | DI 10ml + WM | WM | <24h | 14d | ①② |
| Yao JH 2007 [56] | 80 (40/40) | 50/30 | 42-78  E: 58.2  C: 58.5 | SQI 250ml + WM | WM | <7d | 20d | ①②④ |
| Yu AH 2002 [57] | 56 (28/28) | 34/22 | 42-75  (59.5) | CI 40-60ml + WM | WM | 1-3d | 14d | ①⑥⑨ |
| Yu GF 2019 [58] | 68 (34/34) | 37/31 | 41-78  E: (48.12±1.12)  C: (48.11±1.13) | HQI 20ml + WM | WM | 2-25h | 14d | ③ |
| Zeng GL 2019 [59] | 63 (32/31) | 35/28 | 41-75  E: (59.2±7.3)  C: (60.4±7.8) | CI 80ml + WM | WM | 4-47h | 20d | ①② |
| Zhang BG 2015 [60] | 60 (30/30) | 32/28 | 48-78  E: (64.27±5.82)  C: (66.12±5.46) | SFI 30-60ml + WM | WM | 1-3d | 14d | ①⑩ |
| Zhang FF 2018 [61] | 140 (70/70) | 76/64 | 46-85  (61.7±4.9) | SI 50ml + WM | WM | <24h | 14d | ①②④⑩ |
| Zhang FX 2012 [62] | 68 (38/30) | 45/23 | 58-60  E: 58  C: 60 | SI 30ml + WM | WM | <72h | 20d | ⑩ |
| Zhang RS 2008 [63] | 50 (25/25) | 27/23 | 42-81  E: 62  C: 61 | SI50ml + WM | WM | / | 14d | ②⑥⑨⑩ |
| Zhang Y 2015 [64] | 106 (53/53) | 58/48 | 45-79  E: (58.2±12.4)  C: (56.7±11.9) | SFI 50ml + WM | WM | / | 14d | ①②⑩ |
| Zhao YX 2019 [65] | 60 (30/30) | 37/23 | 40-79  E: (58.64±5.37)  C: (57.78±6.19) | DI 10ml + WM | WM | <6h | 14d | ②③ |
| Zhen HX 2019 [66] | 88 (44/44) | 45/43 | 53-77  E: (68.75±3.56)  C: (67.88±4.51) | SI 20ml + WM | WM | 1-4h | 14d | ①⑩ |

N total number, E experimental group, C control group, M male, F female, min minutes, h hours, d days, SFI shenfu injection, SI shenmai injection, SQI shenqifuzheng injection, HQI huangqi injection, SMI shengmai injection, DI dazhuhongjingtian injection, CI ciwujia injection, WM western medicine. Outcomes: ① clinical efficacy, ② neurological impairment, ③ ADL, ④ TNF-α, ⑤ IL-6, ⑥ FIB, ⑦ HCV, ⑧ LCV, ⑨ PV, ⑩ ADRs. / no report.

**References:**

[1] C. Y. Cao and L. N. Huang, "Analysis the Untoward and Curative Effective of Shenmai Injection Combine With Western Medicine in Treatment of Acute Cerebral Infraction," China Continuing Medical Education, vol. 8, no. 4, pp. 201-202, 2016.

[2] H. J. Chen, W. X. Chen, and X. Li, "Clinical study on Dazhu Hongjingtian Injection for treatment of acute recurrent cerebral infarction," Guangxi Medical Journal, vol. 41, no. 6, pp. 713-716, 2019.

[3] H. Y. Chen, "Effect of edaravone combined with dazhuhongjingtian on acute cerebral infarction and its effect on improvement of cerebral nerve function and serum levels of inflammatory factors," Anti-Infection Pharmacy, vol. 15, no. 10, pp. 1779-1782, 2018.

[4] J. B. Chen, D. R. Zheng, S. A. Wang, and G. H. Sun, "The effect of ciwujia injection on the improvement of endothelial function in patients with acute cerebral infarction," Jiangxi Journal of Traditional Chinese Medicine, vol. 50, no. 443, pp. 45-47, 2019.

[5] S. Chen, B. S. Zhang, and L. Chen, "Effects of dazhuhongjingtian injection on serum SOD, CAT and gsh-px in patients with acute cerebral infarction," Guiding Journal of Traditional Chinese Medicine and Pharmacy, vol. 20, no. 7, pp. 39-41, 2014.

[6] Z. M. Chen and W. B. Liu, "Effects of shenmai injection on erythrocyte immune function, coagulation function and cytokines in aged patients with acute cerebral infarction," Chinese Journal of Gerontology, vol. 38, 2018.

[7] P. R. Cheng, Y. M. Jiang, F. L. He, and J. J. Liu, "Clinical study of shengmai injection in the treatment of acute cerebral infarction," Heilongjiang Journal of Traditional Chinese Medicine, no. 4, pp. 19-20, 2015.

[8] H. Cui, Y. Ma, H. S. Han, Z. Y. Xiang, and P. Y. Ding, "Rhadiola Extract Injection Combined with Polivy in the Treatment of Acute Cerebral Infarction Patients," Progress in Modern Biomedicine, vol. 13, no. 15, pp. 2936-2939, 2013.

[9] X. L. Du, C. H. Jia, X. Qin, et al., "The Clinical Research on Measuring Serum Cholesterol and the Ciwujia Treatment in the Acute Cerebral Infraction," Chinses Journal of Medicinal Guide, vol. 6, no. 6, pp. 431-432, 2004.

[10] B. Fan, "Clinical observation of astragalus injection and low molecular weight heparin in the treatment of ischemic cerebrovascular disease," International Medicine & Health Guidance News, vol. 15, no. 22, pp. 48-51, 2009.

[11] R. M. Fan, "Effect of edaravone combined with rhodiola rosea in the treatment of acute cerebral infarction," Strait Pharmaceutical Journal, vol. 31, no. 4, pp. 143-144, 2019.

[12] X. D. Fei, "Observation on the therapeutic effect of dazhuhongjingtian injection combined with ozagrel sodium in the treatment of acute cerebral infarction," Journal of Practical Traditional Chinese Medicine, vol. 33, no. 12, pp. 1387-1388, 2017.

[13] K. Q. Fu and Y. L. Wang, "Clinical observation of 84 cases of acute cerebral infarction treated by shenmai injection," Zhejiang Journal of Traditional Chinese Medicine, vol. 45, no. 7, p. 543, 2010.

[14] M. Han, "Clinical effect of Shenmai injection in acute cerebral infarction and its effect on hemodynamics and inflammatory factors," Clinical Research and Practice, no. 14, 2018.

[15] T. He, A comparative study on the influence of rhodiola crenulata plus probucol on efficacy and related factors in cerebral infarction, Hebei Medical University, 2017.

[16] Z. C. He and L. M. Hua, "Effects of Radix astragali inoculation fluid on serum TNF-αand ET-1 levels in patients with acute cerebral infarction and significance," Clinical Focus, vol. 25, no. 3, pp. 196-199, 2010.

[17] A. M. Hu and H. W. Xu, "Effect of ciwujia injection on acute cerebral infarction," Chinese Journal of Rural Medicine and Pharmacy, vol. 11, no. 5, pp. 45-46, 2004.

[18] M. Hu, "Effect of Shenmai Injection Combined with Ozagrel on NIHSS Sore and Serum Levels of Hcy and LP (a) in Patients with Acute Ischemic Stroke," The Journal of Medical Theory and Practice, vol. 31, no. 7, 2018.

[19] X. Huang, "Clinical effect of shenqi fuzheng injection on acute cerebral infarction," Hainan Medical Journal, vol. 20, no. 4, pp. 88-89, 2009.

[20] K. J. Jiang, H. W. Mao, and H. F. Pan, "Clinical observation on the treatment of acute cerebral infarction with conventional western medicine and ciwujia injection," Chinese Journal of Integrated Traditional and Western Medicine in Intentire Critical Care, vol. 10, no. 6, p. 384, 2003.

[21] A. H. Li and K. F. Ke, "Effect of shenmai injection on 40 cases of acute cerebral infarction," Medical Journal of Communications, vol. 21, no. 1, pp. 143-144, 2007.

[22] D. X. Li, H. Y. Lu, and W. G. Li, "Treatments of acute cerebral infarction with defibrase and Ciwujia," Chinese Journal of Practical Nervous Diseases, vol. 10, no. 3, pp. 37-38, 2007.

[23] Q. Y. Li, Y. Wang, and G. D. Li, "Effect of astragalus injection on acute cerebral infarction," People's military surgeon, vol. 40, no. 7, p. 427, 1997.

[24] R. H. Li, Z. Y. Guan, S. M. Zong, and Y. G. Shi, "Effect of shenmai injection on acute cerebral infarction," Henan Journal of Practical Nervons Diseases, vol. 5, no. 3, pp. 58-59, 2002.

[25] X. P. Li, "Effect of shenmai injection combined with sodium butylphthalide chloride injection on acute cerebral infarction and its influence on serum inflammatory index," Chinese Journal of Convalescent Medicine, vol. 28, no. 6, pp. 623-626, 2019.

[26] Y. Y. Li and L. Chen, "Clinical Effect of Edaravone Injection Combined with Shenmai Injection in Treating Elderly Patients with Ischemic Stroke and the Impact on Serum Levels of Vaspin and Resistin, and Cerebral Hemodynamics," Practical Journal of Cardiac Cerebral Pneumal and Vascular Disease, vol. 27, no. 10, 2019.

[27] X. L. Liang, W. H. Wang, Q. X. Shi, X. P. Chen, and G. X. Hu, "Clinical Observation on Shenmai Injection Combined with Rosuvastatin Calcium for Acute Ischemic Stroke," Journal of New Chinese Medicine, vol. 50, no. 11, 2018.

[28] Q. K. Lin, X. G. Chen, X. Yang, and H. Z. Zhou, "Clinical observation of shengmai injection in the adjuvant treatment of acute cerebral infarction," Journal of Emergency in Traditional Chinese Medicine, vol. 22, no. 10, pp. 1779-1780, 2013.

[29] D. Liu, S. Zhang, and P. Y. Tang, "Observation on Efficacy of Shenfu Injection Combined with Edaravone Injection in Treatment of Acute Cerebral Infarction," Evaluation and Analysis of Drug-Use in Hospitals of China, vol. 17, no. 10, pp. 1360-1362, 2017.

[30] S. J. Liu, Y. H. Han, and J. Dong, "Clinical observation on the treatment of acute cerebral infarction with sodium ferulate sodium chloride injection in combination with dazhuhongjingtian injection," Medical Journal of National Defending Forces in Northwest China, vol. 37, no. 10, pp. 694-695, 2016.

[31] Z. M. Long, M. Z. Long, L. M. Jiang, and Z. J. Zhang, "Clinical Observation on Effect of Ciwujia Injection in Treating of Acute Ischemic Stroke," Chinese Journal of Information on TCM, vol. 7, no. 9, pp. 37-38, 2000.

[32] R. Lu and P. Yang, "Effects of shenmai injection on serum TNF- and il-6 in patients with acute ischemic stroke," Chinese Journal of Integrative Medicine on Cardio-/Cerebrovascular Disease, vol. 11, no. 11, pp. 1399-1400, 2013.

[33] H. X. Mei, Z. S. Wang, and T. J. Cai, "Clinical observation on treatment of acute cerebral infarction with dazhuhongjinttian injection combined with ozagrel," Journal of Emergency in Traditional Chinese Medicine, vol. 22, no. 3, pp. 484-485, 2013.

[34] C. J. Peng, P. J. Zhong, H. Q. Liu, and S. Q. Mo, "The Clinical Observation on the Effect of Ciwujia Injection (刺五加注射液) on the Level of TXB2 and 6 - Keto - PGF1α of Patients with Cerebral Infarction in Acute Stage," Journal of Emergency in Traditional Chinese Medicine, vol. 15, no. 7, pp. 732-733, 2006.

[35] P. S. Qin, W. L. Zhu, K. Y. Liu, and X. C. Pu, "Investigation on Clinical Effect of Ciwujia Injection and Jiangxianmei Combination for Acute Cerebral Infarction," Chinese Traditional Patent Medicine, vol. 23, no. 7, pp. 500-502, 2001.

[36] L. L. Sha, "Effect observation of dazhuhongjingtian combined with edaravone in the treatment of acute cerebral infarction," Chinese Journal of Practical Nervous Diseases, vol. 16, no. 1, pp. 43-44, 2013.

[37] Y. G. Tang, "Clinical observation of 35 cases of acute watershed cerebral infarction treated by shenmai injection," Zhejiang Journal of Traditional Chinese Medicine, vol. 46, no. 4, pp. 238-239, 2011.

[38] H. Z. Wang, "Clinical observation and nursing intervention of shenmai injection in the treatment of community cerebral infarction," Strait Pharmaceutical Journal, vol. 25, no. 1, pp. 216-217, 2013.

[39] J. M. Wang, "Treatment of 54 cases of acute cerebral infarction with dazhuhongjingtian combined with edaravone," China Pharmaceuticals, vol. 24, no. 4, pp. 73-74, 2015.

[40] J. X. Wang, "Effect of shenmai injection on serum c-reactive protein in patients with acute ischemic stroke," Henan Traditional Chinese Medicine, vol. 34, no. 4, 2014.

[41] L. Wang and Q. B. Guo, "Effects of shenmai injection on hemorheology, cerebral hemodynamics and inflammatory factors in acute cerebral infarction," Chin J of Practical Nervous Diseases, vol. 17, no. 18, pp. 105-106, 2014.

[42] Q. Wang, F. Fang, W. M. Feng, and X. Z. Zhu, "Effects of shenmai injection on hemorheology, cerebral hemodynamics, serum TNF-α and IL-6 levels in patients with acute cerebral infarction," Chinese Journal of Traditional Medicine Science and Technology, vol. 20, no. 3, pp. 224-225+215, 2013.

[43] X. P. Wang, "46 Cases Clinical Observation of the Effect of Rhodiolarosea Combined with Edaravonein Treating Acute Cerebral Infarction," Chinese Journal of Ethnomedicine and Ethnopharmacy, vol. 28, no. 14, pp. 113-115, 2019.

[44] Y. P. Wang and J. F. Zhan, "The Effect of Ciwujia Injection on Angiotensin-Ⅱ and Endothelin in Patients with Acute Cerebral Infarction," Chinese Journal of Integrative Medicine on Cardio-/Cerebrovascular Disease, vol. 4, no. 3, pp. 191-192, 2006.

[45] Z. Y. Wang, "Clinical analysis of 32 cases of acute cerebral infarction treated by acanthopanax," Modern Medicine Health, vol. 21, no. 12, p. 1497, 2005.

[46] Y. L. Wei, "Clinical efficacy of sofren injection combined with cattle encephalon glycoside and ignotin injection in treatment of acute ischemic stroke," China Medical Engineering, vol. 25, no. 3, pp. 16-19, 2017.

[47] P. H. Wu, A. M. Wang, and X. L. Zheng, "Effect of Shenmai injection on hemodynamics in patients with cerebral infarction (hypoperfusion/in-sufficient ability of clearing embolus)," Journal of Shanxi College of Traditional Chinese Medicine, vol. 16, no. 1, pp. 46-47, 2015.

[48] L. L. Xia and Y. Q. Wang, "Rhadiola extract injection combined with sodium ozagrel in the treatment of acute cerebral infarction," Medical Journal of National Defending Forces in Northwest China, vol. 33, no. 5, pp. 518-521, 2012.

[49] L. L. Xia, "Clinical effect analysis of dazhuhongjingtian combined with edaravone in the treatment of acute cerebral infarction," Contemporary Medicine, vol. 19, no. 3, pp. 7-9, 2013.

[50] C. Y. Xiao, C. J. Peng, and J. Z. Pei, "Effects of ciwujia injection on plasma prostacyclin and thrombin in patients with acute cerebral infarction," Guangdong Medical Journal, vol. 28, no. 12, pp. 2024-2025, 2007.

[51] H. B. Xu and C. Y. Zhou, "Clinical observation of 30 cases with acute cerebral infarction treated by shenmai injection," Modern Journal of Integrated Traditional Chinese and Western Medicine, vol. 10, no. 17, pp. 1629-1630, 2001.

[52] L. B. Xu and X. S. Ding, "Effect of edaravone injection combined with shenfu injection on acute cerebral infarction," Chinese Journal of Ethnomedicine and Ethnopharmacy, no. 21, pp. 38-39, 2010.

[53] P. W. Xu and H. M. Pei, "Low molecular weight heparin combined with ciwujia injection in the treatment of 56 cases of acute cerebral infarction," China Pharmaceuticals, vol. 12, no. 5, pp. 66-67, 2003.

[54] F. Xue, "Effect of shenmai injection on 30 cases of acute cerebral infarction," Medical Journal of Communications, vol. 21, no. 6, pp. 659-660+662, 2007.

[55] J. S. Yang, "Clinical efficacy of hongjingtian injection combined with edaravone in the treatment of acute cerebral infarction," China & Foreign Medical Treatment, vol. 35, no. 22, pp. 126-128, 2016.

[56] J. H. Yao, F. Li, L. W. Peng, G. F. Yang, and B. S. Fu, "Effects of shenqifuzheng injection on plasma ET-1 and TNF-α in patients with acute cerebral infarction," Chinese Journal of Medicine, vol. 42, no. 10, pp. 49-50, 2007.

[57] A. H. Yu and L. Lv, "Ciwujia injection in the treatment of 28 cases of acute cerebral infarction," China Pharmaceuticals, vol. 11, no. 8, p. 70, 2002.

[58] G. F. Yu, "Effect of astragalus injection on muscle strength recovery of hemiplegic limbs after acute cerebral infarction," Strait Pharmaceutical Journal, vol. 31, no. 5, pp. 229-230, 2019.

[59] G. L. Zeng, G. Q. Zhao, L. Zhou, and L. Y. Shen, "Effect of ciwujia injection combined with edaravone on acute cerebral infarction," Contemporary Medical Symposium, vol. 17, no. 1, pp. 124-125, 2019.

[60] B. G. Zhang, Y. B. Huang, and X. L. Yang, "The Clinical Observation of Shenfu Injection on Patients with Acute Cerebral Infarction (Deficiency of Vigour and Vitality)," Journal of Emergency in Traditional Chinese Medicine, vol. 24, no. 8, 2015.

[61] F. F. Zhang, Y. Sun, L. Zhang, Q. G. Dong, Z. Y. Chen, and B. Liu, "Clinical study on Shenmai Injection combined with butylphthalide in treatment of acute cerebral infarction," Drugs & Clinic, vol. 33, no. 7, pp. 1635-1639, 2018.

[62] F. X. Zhang, X. L. Huang, T. F. Zhou, and J. M. Zhang, "Shenmai injection in the treatment of 38 cases of acute cerebral infarction," China Pharmaceuticals, vol. 21, no. 22, p. 102, 2012.

[63] R. S. Zhang, "Effect of shenmai injection on hemodynamics in patients with acute cerebral infarction," China Practical Medicine, vol. 3, no. 18, pp. 93-94, 2008.

[64] Y. Zhang, "Effect evaluation of cinepazide maleate combined with shenfu injection in the treatment of acute cerebral infarction," Chinese Journal of Practical Nervous Diseases, vol. 18, no. 23, pp. 114-115, 2015.

[65] Y. X. Zhao, "Effect of dazhuhongjingtian injection combined with alteplase intravenous thrombolysis in the treatment of acute cerebral infarction," Henan Medical Research, vol. 28, no. 3, pp. 515-517, 2019.

[66] H. X. Zhen, "Efficacy analysis of shenmai injection combined with alteplase intravenous thrombolysis in the treatment of acute ischemic stroke patients," Clinical Journal of Diabetes World, vol. 16, no. 11, p. 30, 2019.
